# Isolated tubal torsion: Successful preoperative diagnosis of five cases using ultrasound and management with laparoscopy

**DOI:** 10.4274/tjod.57984

**Published:** 2017-09-30

**Authors:** Erdem Fadıloğlu, Rıza Dur, Erhan Demirdağ, Çağatayhan Öztürk, Şeyma Fadıloğlu, Metin Kaplan, Ömer Lütfi Tapısız

**Affiliations:** 1 University of Health Sciences, Ankara Etlik Zübeyde Hanım Women’s Health Training and Research Hospital, Clinic of Obstetrics and Gynecology, Ankara, Turkey; 2 Ankara Numune Training and Research Hospital, Clinic of Obstetrics and Gynecology, Ankara, Turkey

**Keywords:** Isolated tubal torsion, ultrasonography, whirlpool sign, case series

## Abstract

Our aim was to evaluate the presentation and diagnostic evaluation of patients with isolated tubal torsion and to evaluate the surgical approach to these patients. We also aimed to define the ultrasonographic diagnostic criteria. Five patients with isolated tubal torsion who were admitted to our gynecology department between January 2014 and January 2017 were evaluated and included in this study. All cases were diagnosed through ultrasonographic imaging alone. The preoperative findings of the patients were similar to those described in the literature. No further imaging modality was used for diagnosis and all patients were managed with laparoscopy. The clinical findings and ultrasonographic findings were consistent with literature. It may be difficult to preoperatively diagnose isolated tubal torsion, which is a rare clinical entity. Evaluation of these patients by an experienced sonographer and knowledge of the ultrasonographic findings of isolated tubal torsion may have vital preventive measures.

## INTRODUCTION

Isolated tubal torsion is a very rare entity, reported as 1 in 1.5 million women^(1)^. It is a challenging preoperative differential diagnosis because of its non-specific clinical findings such as lower abdominal pain, nausea, vomiting and fever, and should be kept in mind while approaching patients with abdominal pain^(2^). This report presents five cases of isolated tubal torsion and their successful preoperative diagnosis using ultrasonography (USG) imaging and management with a laparoscopic approach at a tertiary health center within 2 years, and an evaluation of literature findings.

## CASE REPORT

Patients diagnosed as having isolated tubal torsion between January 2014 and January 2017 were included in this presentation after acquiring informed consent from all patients. Five patients with lower abdominal pain at different intensities were diagnosed as having tubal torsion using USG imaging. All five patients had significant acute lower abdominal pain. The patients described a sudden onset of symptoms, and four patients had slightly increased white blood cell counts (WBC). Only case 5 had a normal WBC count. Three of five patients had nausea and vomiting as other symptoms. The decision was made for all patients to undergo surgery with a preoperative diagnosis of tubal torsion with or without coexisting adnexal masses. No other imagining modality beyond USG was used for the preoperative diagnosis. All diagnoses were confirmed under laparoscopy, but coexisting risk factors were misdiagnosed in case 1 and 2, as listed at Table 1. Four of the five patients were managed with salpingectomy, and detorsion was performed in only one patient who desired future fertility. None of the patients had postoperative complications and all were discharged within 48 hours. The important feature of these five patients was the correct preoperative diagnosis acquired using just USG, which was confirmed during the operation, and also through postoperative pathologic examination of specimens.

## DISCUSSION

The potential risk factors for isolated tubal torsion are tubal pathologies such as hydrosalpinx, paratubal cysts or ovarian masses, and altered tubal function. However, normal tubal appearance was mostly found in cases of isolated tubal torsion^(3)^. One of our five cases had normal tubal structure, two had paratubal cysts, and one had hydrosalpinx, consistent with the literature.

The USG features of tubal torsion may vary widely. Preoperative suspicion may rise with an image of elongated, convoluted cystic mass, tapering as it nears the uterine cornua^(4)^. An increased resistance index due to decreased blood flow determined using Doppler USG may also strengthen the suspicion of torsion^(5)^. Nevertheless, abnormal Doppler findings are not a necessity for the diagnosis of torsion. Identifying a normal ipsilateral ovary may strongly suggest tubal torsion.

As in other torsion cases, a systematic evaluation of the adnexal areas is needed in order to be able to identify isolated tubal torsion cases with USG. A systematic evaluation of symptomatic patients should always include evaluation of tubal segments. In the correct evaluation of the adnexal area, the starting point should be the interstitial tubal segment, which is the starting point of the adnexa, and from here onwards, the entire tuba continues to medial to the fimbrial tip. Neighboring structures should also be evaluated at the same sections. The interstitial and trunk part of the uterine tube, the proper ovary ligament (ligamentum ovarii proprium) and the round ligament are located in the cornual area near the uterus. Over-torsions in this region are mostly around the proprium ligament axis, and tubal torsion mostly occurs by rotating around its own axis.

**Figure 1:**
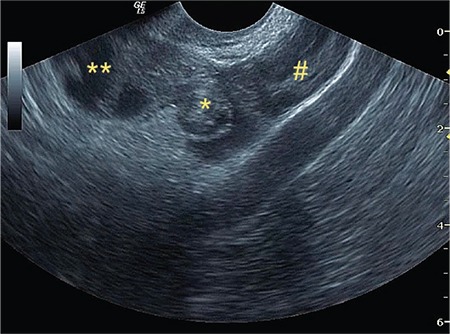
*“Whirlpool” sign, **hydrosalpinx at the distal side of tubal torsion, #ovarian tissue

**Figure 2:**
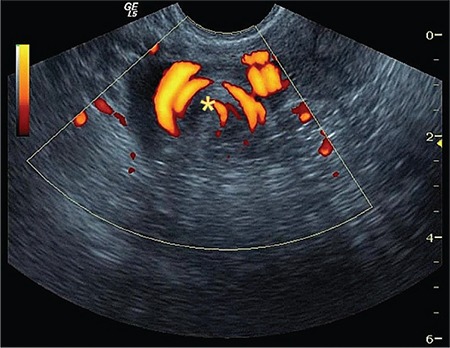
Ongoing circulation within the “whirlpool” sign showing circular pattern

In isolated tubal torsion cases, torsional twist is directly observed on USG imaging (Figure 1). This finding must be seen and seen absolutely for the exact diagnosis of any kind of torsion in all cases. This finding is enough for the diagnosis of torsion because it is the physical image of torsion. Due to circulation problems, the tuba seems edematous and swollen and it is easily distinguishable from the overdose around the hypoechoic irregular soft tissue mass formed by the adnexal inferior tubal edema. When the “whirlpool” finding is evaluated with power Doppler, the venous circulation may be observed in a circular pattern (Figure 2). However, with the deterioration of the arterial circulation by the progress of the tubal torsion, this Doppler “whirlpool” image may disappear, but the sonographic image sign of turning around the axis remains. As the event progresses, the walls of the tuba become thicker with the edema and findings of hydrosalpinx and hematosalpinx in the lumen of the tuba begin to show themselves. Tubal torsion of this size may ultimately be regarded as ovarian torsion because the hematosalpinx is very enlarged and it is difficult to monitor the ovary when it starts to form incomplete septations.

The progression of torsion causes hematosalpinx, tubal rupture, and peritubal hematomas, which become more complicated and harder to diagnose. If the heterogeneous mass forming in the adnexal area is not carefully evaluated, it can easily be confused with a ruptured ectopic pregnancy.

**Figure 3:**
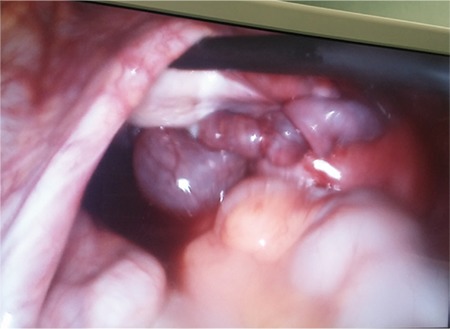
Isolated tubal torsion captured during laparoscopy without any other adnexal pathologies (case 3)

**Figure 4:**
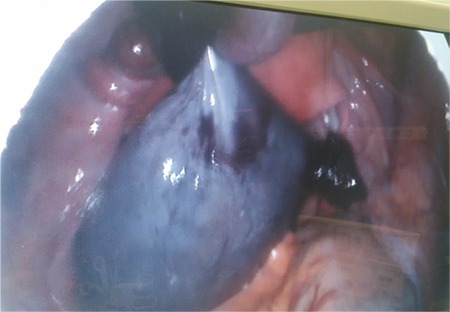
Isolated tubal torsion with concurrent paratubal cyst (case 5)

The primary approach to tubal and ovarian torsion should be laparoscopy and recommended as primary approach^(2)^. Torsions are mostly treated with this approach owing to the advanced accessibility with laparoscopy. We prefer the laparoscopic approach for both ovarian and tubal torsions, as in these 4 cases, which were successfully managed using laparoscopy (Figure 3, 4).

In conclusion, tubal torsion is an emergency condition and a correct preoperative diagnosis should be acquired immediately. Ultrasound criteria for tubal torsion diagnosis require careful evaluation, thus it should only be performed by an experienced practitioner, and laparoscopy should be the primary choice of treatment.

## LITERATURE REVIEW

Isolated tubal torsion is a very rare clinical entity. Correct diagnosis requires great caution and experience because of it is rarity and non-specific symptoms. The most common symptoms were listed as abdominal pain, vomiting, and fever in pediatric case series^(6)^. Also, bowel and bladder problems, lower abdominal mass diagnosed during examination, and elevated WBCs may be other symptoms^(7)^. The etiology remains unclear, but anatomic changes, positional changes, trauma, previous surgeries or gravid uterus are listed as potential risk factors^(8)^. There is also a higher probability for right tubal torsion then left due to the position of the sigmoid colon and slow venous drainage of the right tuba^(9)^. Paratubal or adherent cysts may also play an important role in the etiology according to literature and our findings^(10)^. This situation may be seen in pregnant patients; isolated tubal torsion should be considered as a diagnosis because of difficulties in imaging^(11)^. Magnetic resonance imaging (MRI) may be used with clinical suspicion both in pregnant and non-pregnant patients for differential diagnosis^(9,12)^. MRI may also show the “whirlpool” sign, which is the image of physical torsion of tubas^(13)^.

Diagnosing torsion correctly using USG is also challenging, but very important because of its speed and easy accessibility. Ultrasonographic findings listed in the literature are mostly the same as the criteria used at our clinic. MRI or computed tomography scans may play role in diagnosis, but have disadvantages such as cost, radiation exposure, and potential hazards during pregnancy^(14)^. Prompt diagnosis is also very important due to the possible results of delay such as necrosis^(15)^.

Management of tubal torsion mostly requires surgical intervention. Salpingectomy may be performed if there is no further desire for fertility, but the proper approach should be detorsion of tuba for preserving fertility(14). Urgent intervention should be performed in symptomatic patients and rare results of chronic tubal torsion such as tubal autoamputation should be known^(16)^.

Isolated tubal torsion is a very rare entity and may be misdiagnosed due to its non-specific symptoms. All physicians must know the diagnostic criteria for prompt diagnosis and a proper evaluations should always include the adnexal area and tubas. A systematic approach to the adnexal area as mentioned may diagnose most tubal torsion cases. After proper preoperative diagnosis, tubal torsions may be managed via laparoscopy. In conclusion, the most important point in managing these patients is a correct and rapid diagnosis.

## Figures and Tables

**Table 1:**
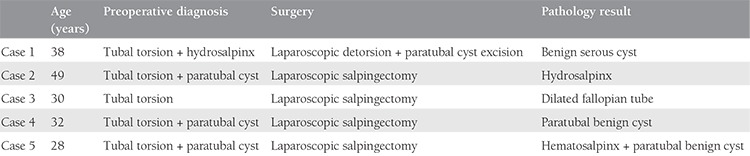
Summary of five cases with preoperative diagnosis and postoperative pathologic results
